# Complete Protection against Pneumonic and Bubonic Plague after a Single Oral Vaccination

**DOI:** 10.1371/journal.pntd.0004162

**Published:** 2015-10-16

**Authors:** Anne Derbise, Yuri Hanada, Manal Khalifé, Elisabeth Carniel, Christian E. Demeure

**Affiliations:** Unité de recherche *Yersinia*, Institut Pasteur, Paris, France; Yale Child Health Research Center, UNITED STATES

## Abstract

**Background:**

No efficient vaccine against plague is currently available. We previously showed that a genetically attenuated *Yersinia pseudotuberculosis* producing the *Yersinia pestis* F1 antigen was an efficient live oral vaccine against pneumonic plague. This candidate vaccine however failed to confer full protection against bubonic plague and did not produce F1 stably.

**Methodology/Principal Findings:**

The *caf* operon encoding F1 was inserted into the chromosome of a genetically attenuated *Y*. *pseudotuberculosis*, yielding the VTnF1 strain, which stably produced the F1 capsule. Given orally to mice, VTnF1 persisted two weeks in the mouse gut and induced a high humoral response targeting both F1 and other *Y*. *pestis* antigens. The strong cellular response elicited was directed mostly against targets other than F1, but also against F1. It involved cells with a Th1—Th17 effector profile, producing IFNγ, IL-17, and IL-10. A single oral dose (10^8^ CFU) of VTnF1 conferred 100% protection against pneumonic plague using a high-dose challenge (3,300 LD_50_) caused by the fully virulent *Y*. *pestis* CO92. Moreover, vaccination protected 100% of mice from bubonic plague caused by a challenge with 100 LD_50_ *Y*. *pestis* and 93% against a high-dose infection (10,000 LD_50_). Protection involved fast-acting mechanisms controlling *Y*. *pestis* spread out of the injection site, and the protection provided was long-lasting, with 93% and 50% of mice surviving bubonic and pneumonic plague respectively, six months after vaccination. Vaccinated mice also survived bubonic and pneumonic plague caused by a high-dose of non-encapsulated (F1^-^) *Y*. *pestis*.

**Significance:**

VTnF1 is an easy-to-produce, genetically stable plague vaccine candidate, providing a highly efficient and long-lasting protection against both bubonic and pneumonic plague caused by wild type or un-encapsulated (F1-negative) *Y*. *pestis*. To our knowledge, VTnF1 is the only plague vaccine ever reported that could provide high and durable protection against the two forms of plague after a single oral administration.

## Author Summary

*Yersinia pestis*, the agent of plague, is among the deadliest infectious agents affecting humans. Injected in the skin by infected fleas, *Y*. *pestis* causes bubonic plague, which occasionally evolves into the very lethal and contagious pneumonic plague. *Y*. *pestis* is also a dangerous potential bioweapon but no plague vaccine is available. The current study describes the development of a vaccine highly efficient against plague in both its bubonic and pneumonic forms. The strategy consists of a live, avirulent, genetically modified *Yersinia pseudotuberculosis* that produces the capsule antigen of *Y*. *pestis*, named F1. The goal was to propose a vaccine that would be both easy to produce rapidly in large amounts with high quality, and easy to administer to individuals via a single oral dose. The VTnF1 strain described fulfills these demands. The immune response generated is long-lasting, involving both antibodies and memory cells directed against F1 and other antigens. We conclude that VTnF1 is a very promising candidate vaccine against plague.

## Introduction

Plague has been one of the deadliest bacterial infections in human history, causing millions of deaths during three major historical pandemics and leaving an indelible mark engraved in human's collective memory. In addition to ancient foci of the disease in Asia and Africa, the last pandemic (plague of modern times), which started one century ago, allowed plague to develop new foci in previously unaffected territories such as Madagascar, Southern Africa, and the Americas. Despite considerable progress in its prevention and cure during the 20th century, plague has recently made a new comeback, causing close to 50,000 human cases during the last twenty years [1], including cases in countries where plague was thought to be extinct [2]. Therefore, plague is categorized by WHO (World Health Organization) as a re-emerging disease [1, 3].

The etiologic agent of plague, *Yersinia pestis*, is a highly pathogenic Gram-negative bacillus, very recently derived from the much less virulent enteropathogen *Yersinia pseudotuberculosis* [4]. Transmission of the plague bacillus to humans generally starts with the bite of an infected flea, causing bubonic plague, the most frequent clinical form of the disease. *Y*. *pestis* occasionally reaches the airways, and the resulting secondary pneumonic plague is highly contagious due to the emission of infected aerosols, causing inter-human transmission of pneumonic plague. This pneumopathy is systematically lethal in usually less than three days if no treatment is administered.

The possible use of the plague bacillus as a bioterrorist weapon is also a serious threat due to its pathogenicity and human-to-human transmission. *Y*. *pestis* has been classified by the Centers for Disease Control (CDC) of the USA among Tier 1 select biological agents. Different strains of *Y*. *pestis* showing resistance to antibiotics currently used to treat patients have been identified in Madagascar [5]. Antibiotherapy can therefore no longer be considered as sufficient against the natural and intentional danger of plague. Facing such a public health risk, vaccines may be one of the only remaining alternatives to limit the death toll in humans. A plague vaccine should confer protection against bubonic plague, the most frequent form of the disease in nature [1], at the origin of pneumonic plague outbreaks. The vaccine should also protect against pneumonic plague, the most contagious and fatal form of the disease.

No plague vaccine is currently licensed. The live attenuated *Y*. *pestis* strain EV76 and its derivatives have previously been used in humans [6, 7], and were found to confer protection. However, the genetic instability of *Y*. *pestis* represents a major obstacle in its use as live vaccine [4, 8]. Several molecular vaccine candidates have been recently developed, among which two molecular vaccines (RypVax^tm^ and rF1V^tm^) are the most advanced in clinical trials [9, 10]. These vaccines rely on a combination of two peptides: the F1 antigen composing the *Y*. *pestis* capsule and the LcrV component of the Type Three Secretion System (TTSS) [9, 10], which are efficient targets of protective immunity against plague [6, 11]. Molecular vaccines are generally adjuvanted with alum, and thus are good inducers of antibody production but poor inducers of cellular immune response [12, 13]. Cellular immunity is, however, important for plague protection [14], and a weak cellular response could explain why F1-V vaccinated African Green Monkeys were poorly protected despite adequate antibody titers [15].

We recently proposed a vaccine strategy against plague based on an oral vaccination with a live, attenuated strain of *Y*. *pseudotuberculosis* [16, 17]. Because this species is a recent ancestor of *Y*. *pestis*, the two species are genetically almost identical, whereas *Y*. *pseudotuberculosis* has much lower pathogenicity and much higher genomic stability [4]. Due to their immunogenicity and antigenic complexity, live vaccines generate both humoral and cell-mediated immune responses without addition of adjuvant, and the response is directed against multiple target antigens, thus inducing an immunological response that could not be circumvented by genetic engineering of *Y*. *pestis*. In addition, a live vaccine, once developed and validated, could easily and rapidly enter mass production, and would be well suited in response to an emergency need. As a proof of concept, we had reported that oral inoculation of two doses of a live, naturally attenuated *Y*. *pseudotuberculosis* could provide 88% protection against bubonic plague [16].

The initial *Y*. *pseudotuberculosis* strain that we tested was not genetically defined [16]. To develop a vaccine strain both avirulent and genetically defined, the virulent *Y*. *pseudotuberculosis* IP32953 strain, whose genome is known [4], was irreversibly attenuated by deletion of genes encoding three essential virulence factors (the High pathogenicity island, YopK, and the pH6 antigen (PsaA [17]). To increase vaccine efficiency, an F1-encapsulated derivative was constructed. This was obtained by cloning the *Y*. *pestis* F1-encoding *caf* operon into a plasmid, and introducing this plasmid into the attenuated V674 *Y*. *pseudotuberculosis*, thus producing the V674pF1 strain. Oral vaccination using 10^8^ CFU protected 100% against pneumonic plague caused by a challenge with 33 LD_50_ *Y*. *pestis* and 80% against a high-dose challenge (3,300 LD_50_) [17]. However, we subsequently found that vaccination with strain V674pF1 protected only 81% of the mice against bubonic plague caused by subcutaneous injection of a moderate dose (100 LD_50_) of *Y*. *pestis*, a level of protection that was judged insufficient. We also observed that production of F1 was not stable and homogenous in the V674pF1 vaccine strain, possibly accounting for the incomplete protection conferred.

The aim of this study was to generate a new vaccine strain that would allow a homogenous production of F1, and to evaluate the immune response elicited and its protective performances against both bubonic and pneumonic plague.

## Methods

### Ethics statement

Animals were housed in the Institut Pasteur animal facility accredited by the French Ministry of Agriculture to perform experiments on live mice (accreditation B 75 15–01, issued on May 22, 2008), in compliance with French and European regulations on the care and protection of laboratory animals (EC Directive 86/609, French Law 2001–486 issued on June 6, 2001). The research protocol was approved by the French Ministry of Research (N° CETEA 2013–0038) and was performed in compliance with the NIH Animal Welfare Insurance (#A5476-01 issued on 02/07/2007).

### Bacterial strains and culture conditions

The *Y*. *pseudotuberculosis* and *Y*. *pestis* isolates used in this study and their derivatives are described in S1 Table [17]. Bacteria were grown at 28°C in Luria-Bertani (LB) broth or on LB agar plates supplemented with 0.002% (w/v) hemin (LBH). Bacterial concentrations were evaluated by spectrometry at 600 nm and plating on LBH or LB plates. Chloramphenicol (Cm, 25 μg/ml), ampicillin (Amp, 100 μg/ml), kanamycin (Km, 30 μg/ml), spectinomycin (Spec, 50 μg/ml), or irgasan (0.1 μg/ml) were added to the media when necessary. All experiments involving *Y*. *pestis* strains were performed in a BSL3 laboratory.

### Cloning of the *Y*. *pestis caf* operon

To introduce the *caf* operon into the *Y*. *pseudotuberculosis* chromosome, the Tn*7* transposition tool was used [18]. First, a mini-Tn*7* containing a Cm resistance cassette was constructed by cloning the Cm-FRT *Kpn*I-fragment from pFCM1 into pUCR6Kmini-Tn*7* digested with *Kpn*I. The resulting plasmid, named pUCR6K-miniTn7-Cm-FRT, was then digested by *Apa*I and *Eco*RI and ligated to the 5 kb-PCR fragment containing the entire *caf* operon from *Y*. *pestis* amplified with primers A (5’-ATAAGAATGAATTCGTGACTGATCAATATGTTGG-3’) and B (5’-CGTTAGGGCCCGTCAGTCTTGCTATCAATGC-3’), which added *Apa*I and *Eco*RI sites at the extremities of the fragment. To insert the *caf* locus into the *Y*. *pseudotuberculosis* V674 chromosome, plasmids pUC18R6KTn*7*-*caf*-Cm^R^ and pTNS2 (transposase provider) were introduced together into V674 by electroporation. Transposants were selected on LB agar plates containing chloramphenicol and verified for their sensitivity to ampicillin. Presence of the transposon Tn*7*-*caf*-Cm^R^ at the chromosomal att-Tn7 site was verified by PCR, using two pairs of primers: A (5’-CACAGCATAACTGGACTGATTTC-3’) and B (5’- GCTATACGTGTTTGCTGATCAAGATG-3’) for the left junction, and C (5’-ATTAGCTTACGACGCTACACCC-3’) and D (5’- ACGCCACCGGAAGAACCGATACCT-3’) for the right junction.

The recombinant *Y*. *pseudotuberculosis* strain containing the Tn*7*-*caf*-Cm^R^ region in its chromosome was initially named V674TnF1, and its use as a vaccine against plague is protected by patent application PCT/IB2012/001609, issued on August 7, 2012. For simplicity, we hereafter refer to this strain as "VTnF1".

To analyze F1 capsule production, bacteria were visualized by optical microscopy in contact with India ink, and an ELISA assay quantifying the F1 capsule on bacteria was performed as previously described [17].

### Construction of the bioluminescent *Y*. *pestis* CO92 Tn7*ail*-*lux*

A mini-Tn*7* transposon containing a Km resistance cassette was constructed by cloning the Km-FRT *Sac*I-fragment from pFKM1 into pUCR6Kmini-Tn*7* digested with *Sac*I. The recombinant pUCR6KTn*7*-Km plasmid was then digested with *Apa*I and *Xma*I, and ligated to the 5878 bp *Apa*I/*Xma*I fragment from pGEN-*lux* (LuxCDABE provider;[19]). The resulting pUCR6KTn*7*-*luxCDABE* plasmid was then digested with *Spe*I and *Xma*I and ligated to the 109 bp promoter region of *ail* (YPO2905), obtained from the *Y*. *pestis* DNA template by PCR with primers E (5'- CGCACTAGTTGGAATACTGTACGAATATCC-3') and F (5'- ataCCCGGGccagattgttataacaatacc). The resulting plasmid was named pUCR6KTn*7*-P*ail-lux*. For transposition of Tn*7*-P*ail*-*lux* into the *Y*. *pestis* chromosome, plasmids pUCR6KTn*7*-P*ail-lux* and pTNS2 were introduced into CO92 bacterial cells by electroporation. Transposants were selected for with LBH agar plates containing Km. Verification of the CO92::Tn7-P*ail-lux* recombinant was performed by PCR using the primer pairs A/B and C/D for chromosomal integration, and by measurement of photon emission using a Xenius plate reader (SAFAS Monaco) for bioluminescence activity. The virulence of the recombinant CO92::Tn7-P*ail-lux* derivative upon s.c. injection was checked and was found to be similar to that of the wild-type strain, with a median lethal dose (LD_50_) of 10 CFU. *In vivo* imaging was performed with an In Vivo Imaging System (IVIS 100, Caliper Life Sciences).

### Animal immunization and in vivo analyses

Mouse vaccinations were performed in a BSL3 animal facility as described previously [17]. Animals were seven-week-old OF1 female mice or (when specified) C57BL/6 mice from Charles River France. Bacterial suspensions (200 μl in saline) were given intragastrically (i.g.) to mice using a curved feeding needle. Animals were monitored for suffering (prostration, ruffled hair) every other day and were weighed to estimate the impact of vaccination. The virulence of the VTnF1 strain by the oral route was tested by infecting i.g. groups of mice (four per dose) with increasing doses of bacteria in order to determine the LD_50_. The LD_50_ value was calculated by the Spearman-Karber method [20]. *In vivo* dissemination of the VTnF1 strain was examined as described previously [17]. Feces (two fecal pellets) were collected from live mice and were homogenized in PBS using disposable homogenizers (Piston Pellet from Kimble Chase, Fisher Sci.). Peyer's patches, spleen, liver and mesenteric lymph nodes were collected aseptically from euthanized mice. They were homogenized in sterile PBS using three mm glass beads and an electric mill (TissueLyser, Qiagen). The bacterial load was determined by plating serial dilutions of the homogenates.

### Plague challenge experiments

Mice were challenged either four weeks or six months after vaccination. *Y*. *pestis* strains were grown at 28°C on LBH plates, and suspensions in saline were prepared for infections. Mice were infected by s.c. injection (100 μl) in the ventral skin on the *linea alba*. The LD_50_ of the CO92Δ*caf* strain by this route was determined by infecting mice (six per dose) with serial dilutions of bacterial suspensions, and was found to be 100 CFU. To induce pneumonic plague, anesthetized mice were infected i.n. as previously described [17] by instillating 10 μl of bacterial suspensions in nostrils (5 μl each). Animal survival was followed for 21 days.

### Evaluation of the immune response

Mouse blood was collected three weeks after vaccination from live animals by puncture of the maxillary artery with a Goldenrod lancet (Medipoint, USA). Microtiter plates (NUNC) were coated either with F1 antigen, or a sonicate of the *Y*. *pestis* CO92Δ*caf* strain (10 μg/ml). The F1 antigen was obtained from *Y*. *pestis* as described previously [21]. The sonicate of the *Y*. *pestis* CO92Δ*caf* strain was obtained by sonication of bacteria grown at 37°C on LB agar as previously described [17]. The Yops antigens were purified as previously described [22], and assays were performed as described before [17]. Briefly, plates were blocked with 5% defatted dry milk and 0.1% Tween 20 in PBS. Sera serially diluted in PBS containing 0.1% BSA were incubated in wells and bound antibodies were detected using horseradish peroxydase (HRPO)–coupled rat antibodies specific for mouse IgG (Bio-Rad). HRPO activity was revealed using TMB substrate (OptiEIA, BD Pharmingen). Antibody (Ab) titers were calculated as the reciprocal of the lowest sample dilution giving a signal equal to two times the background. To analyze immunoglobulin isotypes, horseradish peroxidase (HRP)-coupled probes directed against mouse IgG1, IgG2a, IgG2b and IgM (Caltag) were used. IgG3 were detected using an uncoupled goat antibody (Abcam), and revealed with an HRP-coupled rabbit antibody against goat IgG (BioRad). For Western blotting, *Y*. *pestis* CO92, wild-type and Δ*caf*, S1 Table) were boiled in Laemmli sample buffer (Thermo) and were loaded on 12% acrylamide gel for SDS-PAGE separation using a MiniProtean device (BioRad). Migrated material was transferred from the gel onto a PVDF membrane (Amersham). All subsequent steps were performed following the western immunoblotting protocol recommended by Cell Signaling Technology (USA). Membrane strips were incubated overnight in pooled 1/100 diluted sera from naïve mice, or mice vaccinated one month earlier. Bound IgG were revealed using a secondary goat anti-mouse IgG coupled to horseradish peroxidase (BioRad). ECL Plus kit (Pierce) was used for peroxidase revelation, and membranes were photographed using a ChemiDoc apparatus (BioRad).

To evaluate the cellular memory in vaccinated animals, splenocytes were cultured as described previously [17]. Briefly, spleens from euthanized animals were dissociated and erythrocytes were lyzed using Gey’s hemolytic solution [23]. Cells extensively washed with cold PBS were resuspended in RPMI 1640 + Glutamax (Invitrogen) supplemented with 5% fetal bovine serum, penicillin + streptomycin, and 10 mM ß-mercaptoethanol. Cells (5x10^6^/condition) were stimulated with either a sterile *Y*. *pestis* CO92Δ*caf* sonicate (5 μg/ml), sterile F1 antigen (5 μg/ml), or Concanavalin A (1 μg/ml; Sigma) as a positive control. After three days, the supernatant was collected and the cytokine content was determined using IFNγ, IL-1β, IL-10, and IL-17 assays (Duosets, R&D Systems).

### Statistical analyses

The Log-rank (Mantel-Cox) test was used to compare survival curves. Unpaired Mann-Whitney or Student's t tests were used to compare bacteria numbers, animal weights, antibody titers, and cytokine production. The paired Mann-Whitney test was used for bioluminescence results. Analyses were performed with Prism 6.0 software (GraphPad Software).

## Results

### Construction of a *Y*. *pseudotuberculosis* strain stably producing the F1 capsule

**Fig 1:**
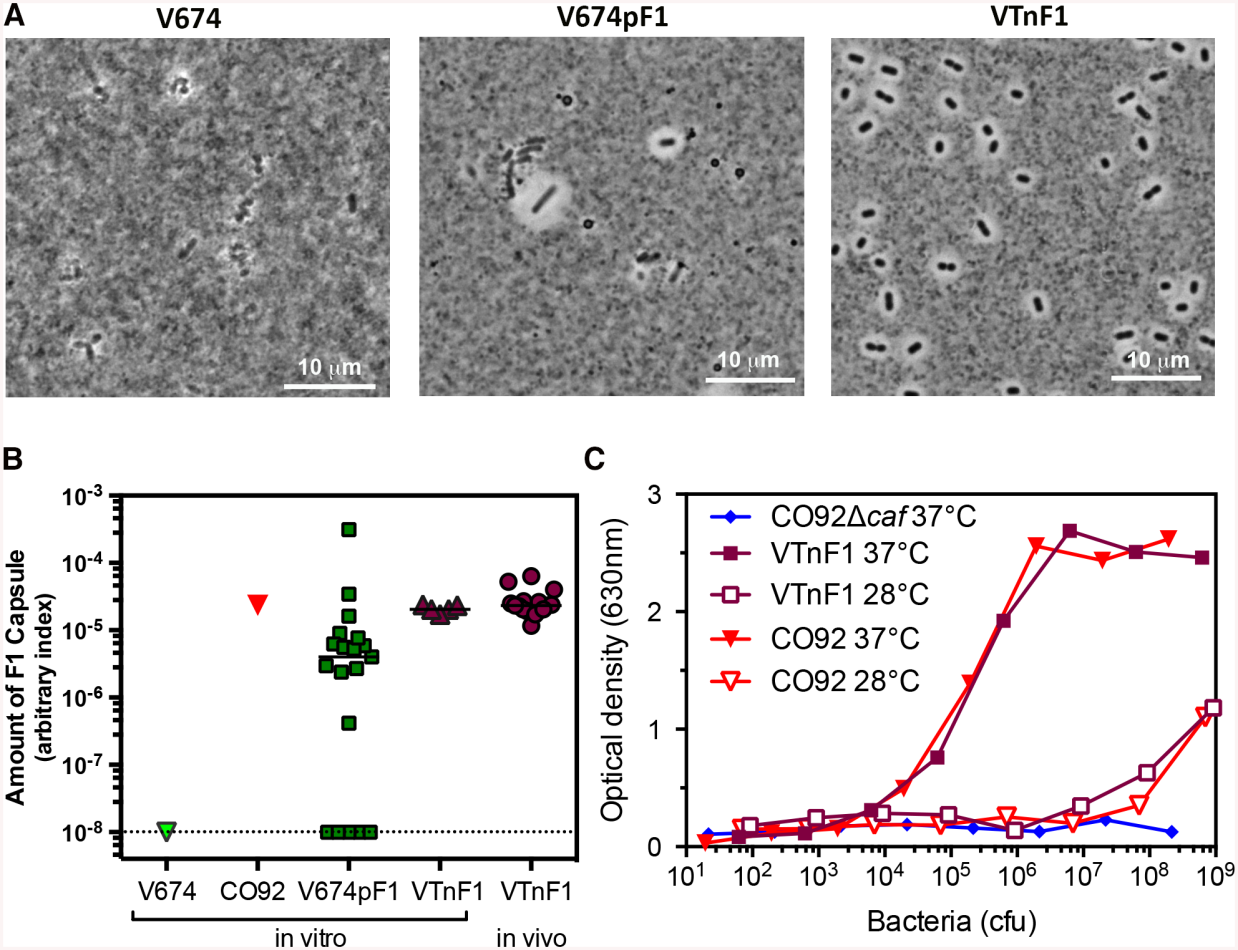
F1 capsule production. (A) V674, V674pF1, and VTnF1 bacteria grown at 37°C were re-suspended in India ink and observed by microscopy. (B) To evaluate the stability of F1 production, isolated colonies of *Y*. *pestis* CO92, or *Y*. *pseudotuberculosis* V674pF1, and VTnF1 were obtained after three subcultures in vitro. Isolated VTnF1 colonies were also obtained by culturing an homogenate of Peyer’s patches taken from mice previously inoculated with VTnF1 (10^8^ CFU i.g.; noted "in vivo"). Serial dilutions of bacteria were tested using an F1-specific ELISA and the F1 index shown was calculated as 1/CFU yielding DO_450nm_ = 1 in the ELISA. The un-encapsulated V674 was used as a negative control. (C) F1 production by VTnF1 was compared to that of *Y*. *pestis* CO92 after growth at 28°C or 37°C, using an F1-specific ELISA, and CO92Δ*caf* was used as negative control.

Strain VTnF1 was constructed by inserting the *caf* operon encoding F1 [17] into the chromosome of the attenuated *Y*. *pseudotuberculosis* V674, using mini-Tn*7* transposon technology [18]. F1 capsule production by recombinant VTnF1 grown at 37°C in LB broth was tested using an F1-specific rapid dipstick test [21], which was clearly F1 positive (S1 Fig). Microscopic visualization of VTnF1 in India ink revealed that all VTnF1 bacterial cells produced the F1 capsule, as visualized by the repulsion of ink particles with comparable thickness (Fig 1A). This contrasted with V674pF1 cultures, which displayed encapsulated and non-encapsulated bacteria. To quantify F1 capsule production, isolated colonies obtained after growth in LB broth at 37°C were tested using an F1-specific ELISA. All VTnF1 colonies were F1 positive and their F1 levels were homogenous (Fig 1B), whereas V674pF1 colonies exhibited various levels of F1 at their surface, indicating both heterogeneity and instability of F1 production. VTnF1 produced as much F1 as *Y*. *pestis*, and this production was temperature-dependent (Fig 1C).

Stability of F1 production was additionally tested after VTnF1 growth in vivo in mice. To this aim, VTnF1 was injected i.g. to animals, and five days later, bacteria were recovered from Peyer’s patches. All VTnF1 colonies were positive for F1 by ELISA (Fig 1B). F1 production was homogenous and comparable to F1 levels of colonies obtained after in vitro culture. This demonstrates that VTnF1 stably and homogenously produced F1 after growing in vivo in mice.

### VTnF1 is rapidly cleared from orally inoculated mice

The V674 strain used to construct VTnF1 was strongly attenuated after intragastric inoculation into mice (LD_50_ >10^10^ CFU;[17]). VTnF1 exhibited an attenuation of virulence similar to V674 and the previous vaccine V674pF1, with an LD_50_ >10^10^ CFU. Mice vaccinated with VTnF1 at a dose of 10^8^ CFU presented transient signs of infection (ruffled hair), and a slight delay in weight gain from day three to seven post-vaccination (average 1.7 g), but they recovered a normal weight from day 10 (S2 Fig). The F1 capsule is dispensable for *Y*. *pestis* virulence in many mouse strains [24–27], but not in others such as C57BL/6 [28]. When a high dose of VTnF1 (4x10^9^ CFU) was inoculated orally to C57BL/6 mice (N = 7), no lethality was observed during the three weeks of follow-up. This confirmed that VTnF1 was strongly attenuated, even for C57BL/6 mice that are more susceptible to F1 action.

**Fig 2:**
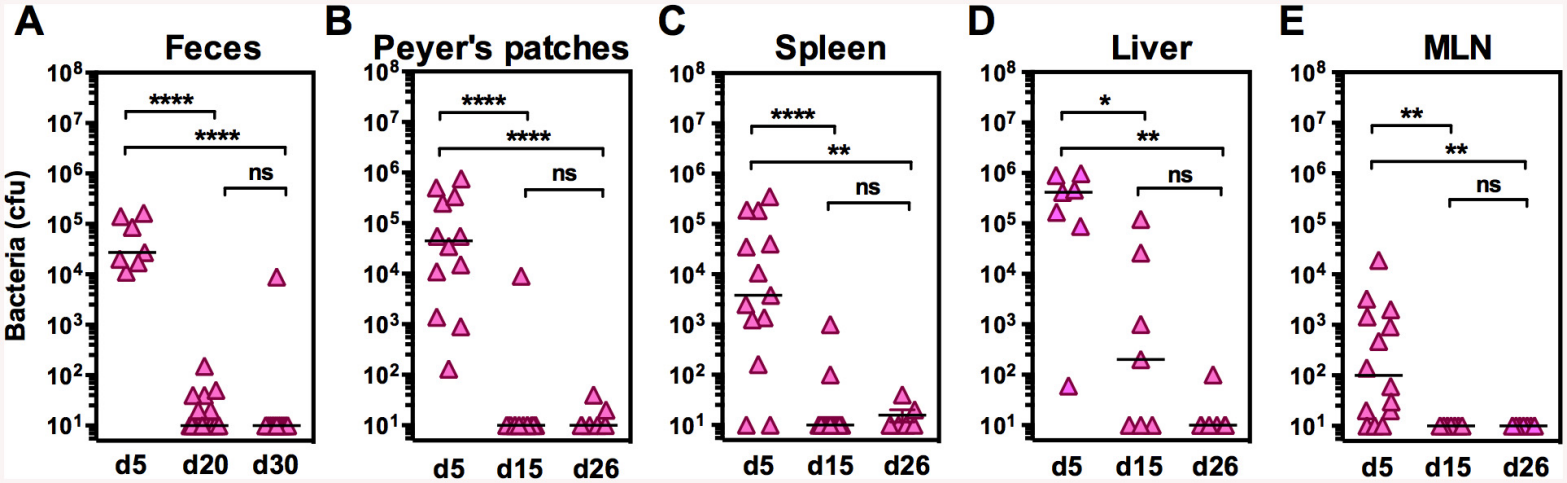
In vivo dissemination of VTnF1 after oral vaccination. Groups of mice were inoculated orally with the VTnF1 vaccine (10^8^ CFU) and were sacrificed at the indicated times to evaluate the VTnF1 loads in: (A) feces (two pellets/mouse), (B) Peyer’s patches (two patches/mouse), (C) the spleen (whole organ), (D) the liver (whole organ), and (E) mesenteric lymph nodes (all). Samples were minced and dilutions were plated on selective agar plates containing kanamycin to count colonies, with a detection limit of 10 CFU/sample. Shown are individual values from 7–14 mice per condition. The horizontal line indicates the median. The Mann Whitney test was used for statistical analysis: *: p ≤0.05, **: p <0.01, ***: p<0.001.

After oral inoculation of VTnF1 (10^8^ CFU), the bacteria were detected on day five-six in the feces and Peyer's patches of all mice (Fig 2A and 2B), and in the spleen, liver and mesenteric lymph nodes (MLN) of most animals (Fig 2C and 2E). However, bacteria were almost completely cleared from the spleen, Peyer's patches and MLN after 15 days, and from the liver after 26 days. Feces tested monthly during the following 5 months remained negative, indicating a vaccine clearance from visceral organs and the gut lumen.

### The humoral immune response elicited by VTnF1 targets multiple antigens, including F1, and is long-lasting

**Fig 3:**
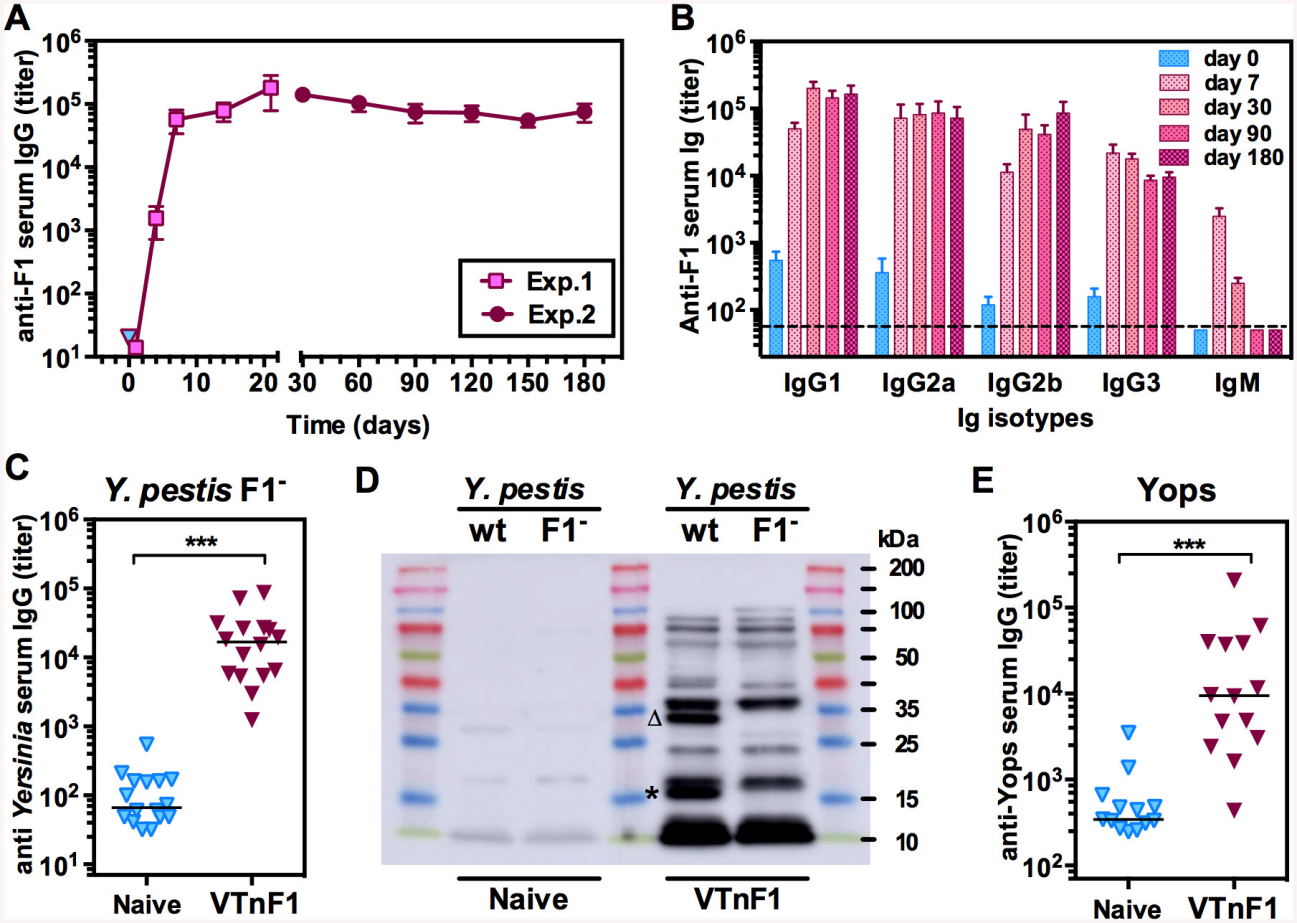
Humoral immune response induced by vaccination. (A) The antibody production induced by VTnF1 vaccination (10^8^ CFU) was quantified at various days post vaccination using an ELISA measuring IgG directed against purified F1 antigen. Shown are the means ± sem of titers observed in two experiments performed during the first month post-vaccination (Exp.1, 7 mice) and during the six-months post-vaccination period (Exp.2, 14 mice). Mean IgG titers at each time point were compared statistically to that on day zero using the Mann-Whitney test, and all were significantly higher (p<0.001), except those on day 1. (B) The different Ig mouse isotypes present in sera were analyzed at sequential times. (C) The IgG response against antigens other than F1 was analyzed by ELISA against a *Y*. *pestis* CO92Δ*caf* sonicate, and (D) by western blotting. The *Y*. *pestis* CO92 wild type or CO92Δ*caf* (noted F1-) were used as source of blotted antigens and sera pooled either from naive mice or mice vaccinated 30 days earlier (noted VTnF1). The Caf1 band is indicated by an asterisk and the Caf1M band by a triangle. (E) The response against purified Yops was also analyzed by ELISA. Shown are results from 14 individual animals (dots), and group medians (horizontal line). The Mann-Whitney test was used for statistical analysis: ***: p<0.001.

The humoral immune response elicited by vaccination with VTnF1 (10^8^ CFU orally) was first evaluated by quantifying serum antibodies against purified F1 at different times post-vaccination over a period of six months. Anti-F1 IgG were detectable as early as four days after vaccination, and reached plateau values after 7 days (Fig 3A). They then maintained at high levels without significant evolution, as shown by the fact that titers observed after six months (d180) were not significantly different from those at d30 (p = 0.08, 14 mice per group). Analysis of the immunoglobulin isotypes revealed that both IgG1, IgG2a, IgG2b and IgG3 contributed to this humoral response, whereas IgM peaked rapidly after vaccination and then fell to low levels (Fig 3B).

IgG recognizing *Y*. *pestis* antigens other than F1 were quantified by ELISA using a sonicate of *Y*. *pestis* CO92Δ*caf* as target. All vaccinated mice had high amounts of IgG against *Y*. *pestis* CO92Δ*caf* antigens (Fig 3C). Western blotting analysis (Fig 3D) confirmed that in addition to F1, at least 12 target antigens were recognized by immune sera. Because conformational epitopes were lost due to the denaturing conditions used for electrophoresis, actual targets were probably more numerous. Among antigens strongly recognized are two components of the Caf operon (absent from the CO92Δ*caf* strain, Fig 3D): the Caf1 and Caf1M antigens (MW 15.6 and 28.7 kDa respectively). Finally, IgG against purified Yops were also measured because these molecules are essential *Y*. *pestis* virulence factors. Such IgG were observed in almost all mice, but with varying levels (Fig 3E). Altogether, our results indicate that the humoral immune response developed rapidly after a single-dose vaccination and involved all main IgG isotypes. The antibodies recognized several antigens other than F1 and were maintained at high levels for extended periods of time without recall vaccination.

### VTnF1-induced memory cells directed toward F1 and other antigens display a pro-inflammatory functional profile

**Fig 4:**
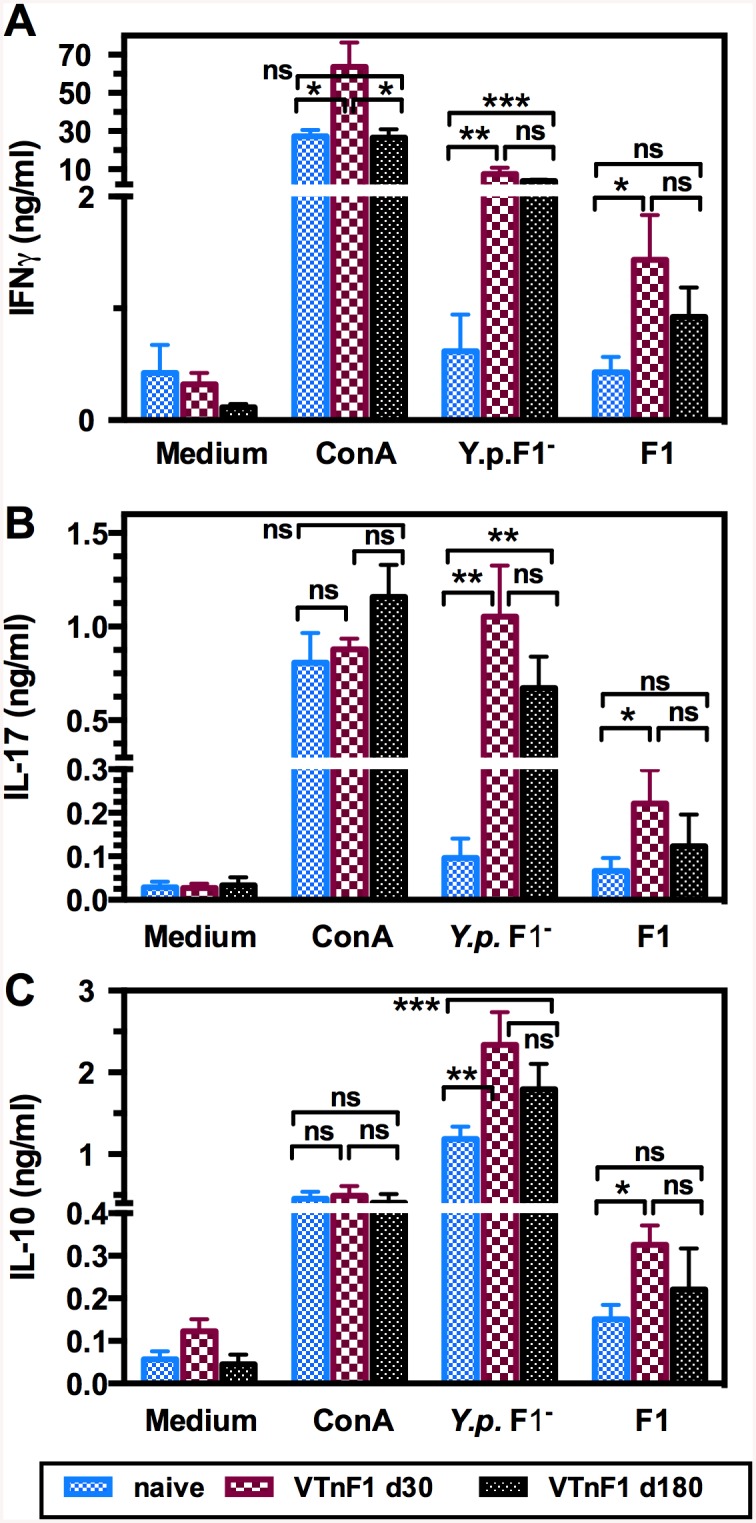
Cellular immune response of vaccinated mice. Splenocytes isolated from mice vaccinated orally with VTnF1 (10^8^ CFU) 30 days (dark red boxes), or 180 days (black boxes) earlier, or from unvaccinated (naive) mice (blue boxes), were stimulated in vitro with 5 μg/ml of either an antigenic preparation of *Y*. *pestis* CO92Δ*caf* (noted *Y*.*p*.F1^-^) or purified F1 antigen, or the mitogen Concanavalin A (ConA, 1 μg/ml) as positive control. Supernatants taken three days after stimulation were tested for the presence of IFNγ (A), IL-17 (B), and IL-10 (C) by ELISA. Shown are the mean ± s.e.m. of 14 mice per condition (two pooled experiments). The unpaired Mann Whitney test was used for statistical analysis: *: p<0.05, **: p<0.01, ***: p<0.001, ns: not significant. Comparable results were obtained in two other experiments using naive and 30-days vaccinated mice (16 per group).

To evaluate the antigen-specific T cell memory elicited by VTnF1, splenocytes taken from vaccinated animals were re-stimulated in vitro with either purified F1 antigen, or with a sonicate of the un-encapsulated *Y*. *pestis* CO92Δ*caf*. Cells from mice vaccinated one month earlier with VTnF1 produced IFNγ, IL-17, and IL-10 in response to F1 (Fig 4). Although these levels were low, they were significantly higher than those of control mice (Fig 4), indicating that vaccination mobilized F1-specific memory T cells producing both pro- (IFNγ, IL-17) and anti-inflammatory (IL-10) cytokines. IL-1β production was also measured, but levels were very low (<0.02 ng/ml) in all conditions.

In contrast, cells from VTnF1 vaccinated mice produced high amounts of IFNγ and IL-17 in response to non-F1 *Y*. *pestis* antigens, reaching levels at least 10 times higher than those observed for cells from naive mice (Fig 4A and 4B). Strikingly, levels of IL-17 were comparable to those induced by the mitogenic lectin ConA, used as a positive control, indicating that a large proportion of responding splenocytes of vaccinated animals recognized *Yersinia* antigens and displayed potent pro-inflammatory functions. This cellular response was also much higher than that stimulated by the F1 antigen alone, reflecting the mobilization of T cells directed against multiple *Y*. *pestis* antigenic targets.

*Y*. *pestis* antigens induced production of IL-10 by splenocytes from both naive and vaccinated mice, probably due to the response of innate immunity cells such as macrophages. However, splenocytes from vaccinated mice produced significantly higher levels of IL-10 than cells from naive mice stimulated with both F1 and other antigens, revealing the recall response of *Y*. *pestis*-specific memory cells with anti-inflammatory activity (Fig 4C).

To evaluate the durability of the cell-mediated response, splenocytes from mice vaccinated six months earlier were also tested. Levels of IFNγ, IL-17 and IL-10 in response to F1 and non-F1 antigens were lower on day 180 than on day 30 post-vaccination, but this difference was not statistically significant. Thus, although slightly reduced, the cell-mediated memory persisted after six months.

### VTnF1 confers high protection against both bubonic and pneumonic forms of plague

**Fig 5:**
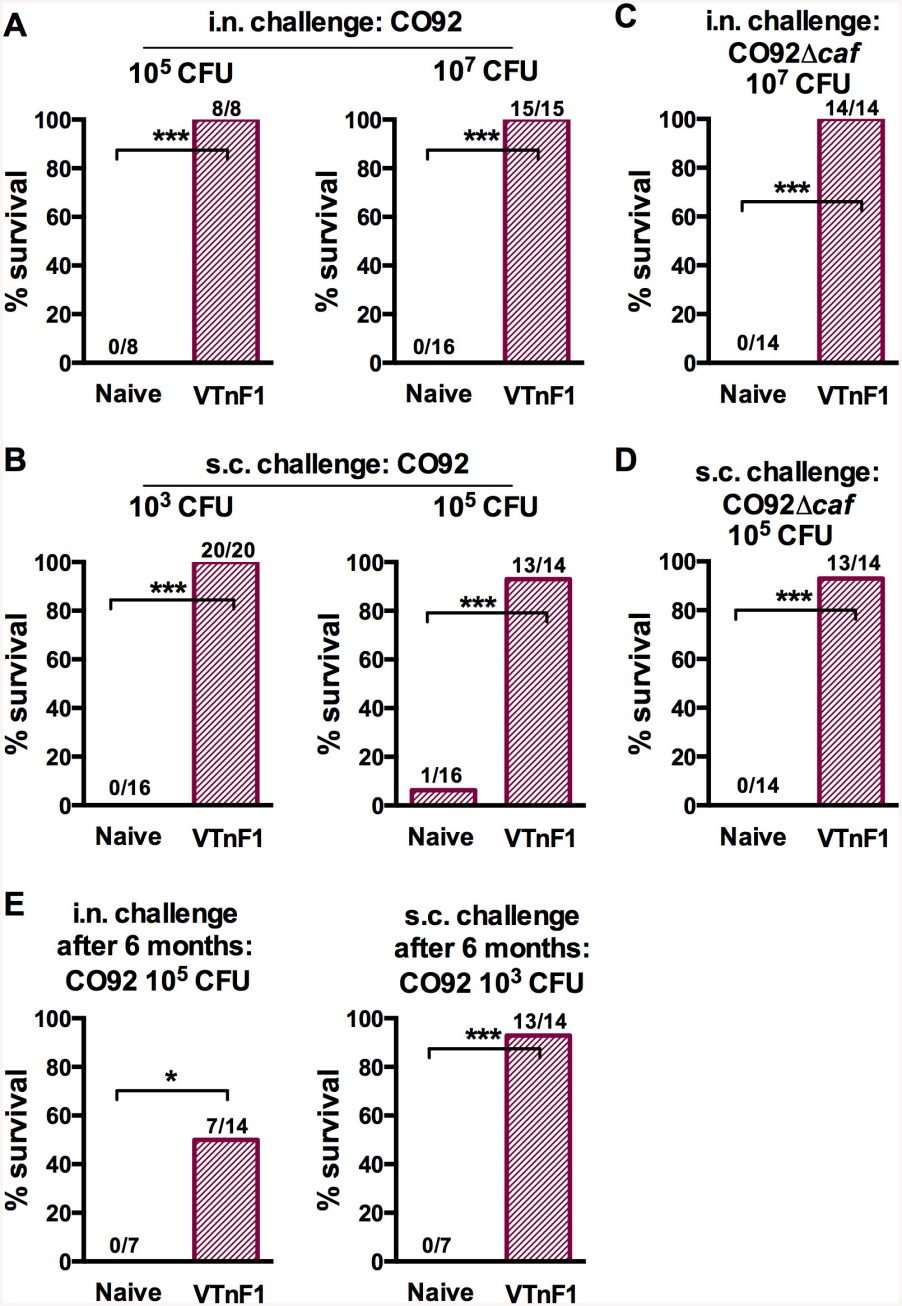
Protection against bubonic and pneumonic plague of mice vaccinated with VTnF1. Mice having received a single oral dose of VTnF1 (10^8^ CFU) were challenged via i.n. or s.c. routes, with various doses of bacteria as indicated. (A-D) Animals were challenged four weeks after vaccination by injection of (A, B) *Y*. *pestis* CO92, or (C, D) the un-encapsulated CO92Δ*caf*. (E) Vaccinated mice were challenged six months after vaccination. Mouse survival was recorded daily for 21 days. The number of mice surviving / number of animals tested is indicated above the corresponding bar for each condition. The Fisher Exact test was used for statistical analysis: *: p≤0.05; ***: p<0.001.

To determine the protective efficacy of VTnF1 against pneumonic plague, vaccinated mice were challenged intranasally with the fully virulent *Y*. *pestis* CO92 strain four weeks after a single oral vaccination (10^8^ CFU of VTnF1). 100% of mice challenged with 10^5^ CFU (33 LD_50_) of *Y*. *pestis* CO92 survived (Fig 5A). Vaccination also protected 100% of the animals exposed to an extremely severe challenge with 10^7^ CFU CO92 (i.e. 3,300 LD_50_; Fig 5A), whereas the previous V674pF1 vaccine strain protected only 80% of the mice infected with this dose [17]. VTnF1 thus appears more protective.

To evaluate the protective efficacy of VTnF1 against bubonic plague, vaccinated mice were infected s.c. with *Y*. *pestis* CO92, four weeks after vaccination. A single oral dose (10^8^ CFU) of the VTnF1 vaccine protected 100% of the mice against 10^3^ CFU (100 LD_50_) of CO92 (Fig 5B). Compared to V674pF1 which only protected 81% (13/16) of animals against bubonic plague in these conditions, VTnF1 was again more protective. When animals vaccinated with VTnF1 received a very severe challenge by s.c. injection of 10^5^ CFU CO92 (10000 LD_50_), 93% of mice (13/14) were still protected.

Because the immune memory is known to decline with time, the protection conferred by VTnF1 was evaluated six months (one third of an OF1 mouse's lifespan [29] after a single-dose oral vaccination with VTnF1. VTnF1 was completely undetectable in mice's feces at day 30 post vaccination and the following months. Upon s.c. challenge with 100 LD_50_ of *Y*. *pestis* CO92, 93% of the animals were still protected against bubonic plague. Furthermore, 50% of the mice survived an i.n. challenge with 33 LD_50_ of *Y*. *pestis* CO92 six months after vaccination (Fig 5D). This indicates that the immune memory elicited by the vaccine persisted and provided a long-lasting protection.

The capacity of VTnF1 vaccination to confer protection against bubonic plague caused by a non-encapsulated *Y*. *pestis* was also evaluated by challenging vaccinated mice with *Y*. *pestis* CO92Δ*caf*. A single oral dose of VTnF1 conferred 100% protection against a severe intranasal challenge with 10^7^ CFU of *Y*. *pestis* (10^7^ CFU, i.e. 3,300 LD_50_, Fig 5C). The same vaccination protected 93% of vaccinated mice against a severe s.c. challenge with 10^5^ CFU (10^4^ LD_50_; Fig 5D). Thus, VTnF1 conferred a strong protection against *Y*. *pestis* in the absence of the F1 pseudocapsule, indicating that, even if plague was caused by a natural or genetically modified F1-negative *Y*. *pestis*, vaccination with VTnF1 would provide a high level of protection.

### Vaccinated mice rapidly control *Y*. *pestis* systemic spread

**Fig 6:**
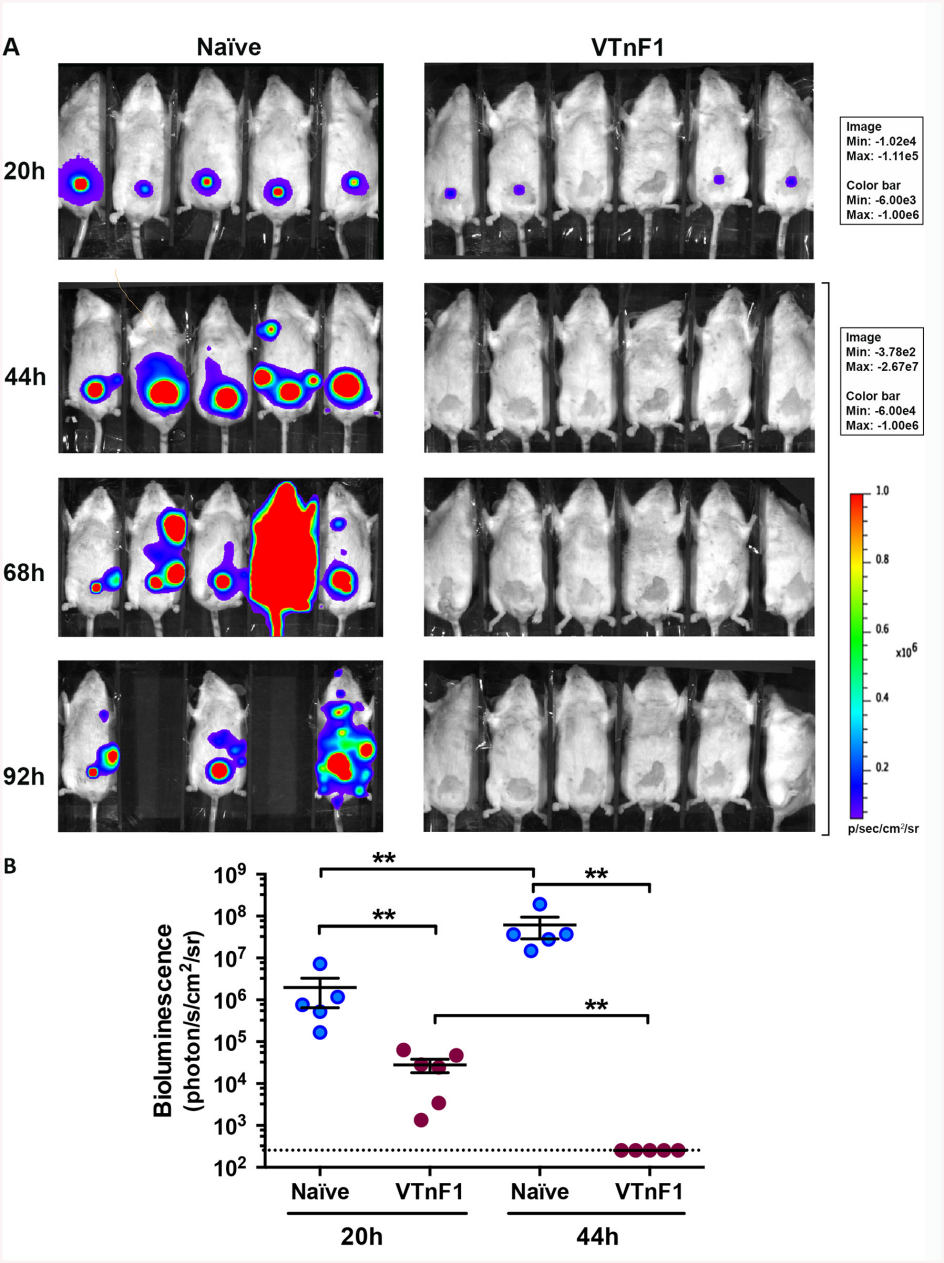
Vaccination with VTnF1 prevents *Y*. *pestis* dissemination from the site of infection. (A) Mice vaccinated with VTnF1 (10^8^ CFU i.g.) were infected four weeks later by s.c. injection of 10^3^ CFU of the bioluminescent *Y*. *pestis* CO92::Tn7-P*ail*-*lux* in the ventral skin. Whole body bioluminescence of the animals was recorded at regular time intervals using an IVIS camera. Shown are white light photographs merged with the bioluminescence signal, scaled in the right margin. (B) The bioluminescence intensity emitted at the site of injection was measured for each animal and shown are means ± s.e.m. for each group. Animals missing at time point 92h died from infection before this bioluminescence recording. The paired Mann-Whitney test was used for statistical analysis: **: p<0.01.

To determine the stage of the infectious process at which immunity provided by VTnF1 controls *Y*. *pestis* proliferation, infection by a bioluminescent *Y*. *pestis* strain (CO92 Tn7*ail*-*lux*) was followed in vivo in live mice. The strain carries in its chromosome the *lux* operon under the control of the *ail* promoter, known to be very active during bubonic plague [30]. In unvaccinated animals, *Y*. *pestis* multiplied at the site of injection and spread to other organs, causing the death of two mice out of five at 92h post-injection. (Fig 6A). In VTnF1-vaccinated mice, the bioluminescence signal was much lower after 20 hours at the site of injection (Fig 6B) and after 44 hours it was no longer visible. Dissemination outside of the injection site was not observed in vaccinated mice. Therefore, vaccination induced fast-acting immune mechanisms that prevented the dissemination of *Y*. *pestis* from the site of injection.

## Discussion

To meet the demand for a vaccine able to confer protective immunity against both bubonic and pneumonic plague, we previously constructed the genetically modified *Y*. *pseudotuberculosis* V674pF1 candidate plague vaccine, which produces the *Y*. *pestis* F1 antigen [17]. Although this vaccine protected against inhalational exposure to *Y*. *pestis*, protection against bubonic plague was not complete. This lack of full protection was potentially explained by the observation that production of the F1 antigen was unstable. The objective of the present work was therefore to improve the vaccine by generating a new strain with improved robustness and efficiency.

Loss of F1 production by V674pF1 mainly resulted from plasmid instability. Others who used *Salmonella* as receiver of the *caf* operon had to apply a sustained antibiotic pressure in vitro to ensure plasmid persistence, but this pressure could not be maintained in vivo [31]. Here, the *caf* operon was transposed into the chromosome [18], and we show that production of F1 capsule by VTnF1 was comparable to that of *Y*. *pestis*, whereas F1 production by V674pF1 was much more heterogeneous and unstable. The stability of VTnF1 characteristics will allow the large-scale production of the live vaccine according to good manufacturing procedures.

VTnF1 provides high protection against both bubonic and pneumonic plague, and is more efficient than V674pF1 used at the same dose (10^8^ CFU) and tested in the same conditions [17]. We had previously reported that only 80% of the mice vaccinated with V674pF1 survived a high-dose pneumonic plague challenge (10^7^ CFU CO92), or a moderate bubonic plague challenge (10^3^ CFU). In contrast, VTnF1 provided complete protection against commonly used bacterial challenges, and almost complete protection against very high challenges. Because V674pF1 and VTnF1 are both derivatives of the V674 attenuated strain, the only possible explanation for this difference of protection is the more homogenous and sustained production of the F1 pseudocapsule by VTnF1. The F1 antigen can activate macrophages [32], an adjuvant effect favorable to the adaptive immune response. Therefore, the efficiency of VTnF1 may result from a stronger stimulation of macrophages, and possibly also of dendritic cells which belong to the same lineage, thus fostering immunity more efficiently.

The high-level protection observed is especially remarkable as it was obtained with a single oral dose of vaccine. Such a very simple procedure is a key advantage as compared to the repeated injections required by most vaccines to confer a protection extended in time. Difficult to perform in the field, repeated injections are considered by public health authorities as a limitation for mass vaccination.

Mice vaccinated with VTnF1 very rapidly control *Y*. *pestis* at the skin entry site. This suggests that easily mobilizable effectors such as antibodies and phagocytes reach the infected tissue. Antibodies might play an essential role in protection conferred by VTnF1, as suggested by the high antibody titers of vaccinated mice. VTnF1 displays much more antigenic diversity than plague molecular vaccines currently under development, which are composed of only F1 and LcrV antigens. The immune response induced by VTnF1 targets various *Y*. *pestis* proteins in addition to Caf1 and Caf1M antigens. This target diversity is valuable to protect against bacteria, which can lose target antigens via gene deletions, as observed for the Caf1/F1 antigen [33]. The anti-F1 IgG are known to provide protection by opsonizing *Y*. *pestis*, facilitated by F1 abundance and surface localization [25, 26, 34]. Whereas the abundant anti-F1 IgG induced by VTnF1 probably play a central protective role against wild type (F1-encapsulated) *Y*. *pestis* [25, 27, 34, 35], the resistance of vaccinated mice to plague caused by an F1-negative *Y*. *pestis* strain demonstrates that the antibodies directed against other antigens also contribute to protection.

*Y*. *pestis* F1-specific IgG induced by VTnF1 are detectable as early as four days after vaccination, a fast kinetic comparable to that observed after vaccination with soluble, recombinant F1 [36]. This prompt onset of IgG production indicates that VTnF1 rapidly interacts with lymphoid cells, probably in Peyer's patches, mesenteric lymph nodes and spleen where VTnF1 was observed. The fast switch of the humoral response from IgM toward antibodies of isotypes IgG1, two and three, generally of high affinity, indicates a strong T-cell dependent response. The presence of abundant IgG3 also indicates that carbohydrate targets are recognized [37]. This diversity is favorable for opsonization via all Fcγ receptors and suggests the involvement of the various B-cells subtypes to the vaccine-induced response [38]. Because IgG levels escalate during the first week to reach the plateau levels observed during the following six months, their contribution to protection against plague at day 30 could already be available at day seven.

Cellular immunity plays an important role against plague [39, 40] and performs critical protective functions during humoral defense against pneumonic plague [41]. Molecular vaccines adjuvanted with alum are poor inducers of this part of the immune response [12, 13]. In contrast, VTnF1 triggered a strong cell-mediated response without adjuvant. Part of the recall cell response was directed against F1, but the most important part was directed toward non-F1 antigens. Its intensity, with IL-17 comparable to a mitogenic stimulation by Concanavalin A, indicates the engagement of a high percentage of splenocytes that are likely recognizing multiple antigenic targets. Memory cells produced IFNγ, IL-17, and IL-10, composing a mixed Th1-Th17 profile. An IFNγ-dependent type 1 immune response is essential for vaccine-induced protection against plague [39]. IFNγ derived from memory T cells instruct potent innate cell activation, resulting in a fast protective immunity against invading microorganisms [42]. IL-17 producing T lymphocytes (Th17 cells) are essential to cure pneumonic plague [43] due to the essential role played by IL-17 in the induction of antimicrobial peptides and attraction of polymorphonuclear leukocytes [44]. The memory response induced by VTnF1 also involves production of the anti-inflammatory cytokine IL-10, which balances potentially harmful effects of IL-17 [45]. In addition to these direct functions, T cells play an important role in antibody-dependent immunity [40] and thus potentiate the humoral response. Altogether, the immune response induced by VTnF1, by combining humoral and cellular mechanisms, has the characteristics required to efficiently clear *Y*. *pestis*.

The sustained humoral and cellular immunity six months after vaccination is unlikely to result from a prolonged stimulation of immunity by live bacteria because VTnF1 was undetectable in feces and organs of most mice one month after vaccination. IgG could be produced by long-lived plasma cells, which differ from so-called memory B cells, and produce abundant IgG without re-stimulation [46]. VTnF1 could have persisted in the gut after one month, for example by durably colonizing the cecum as recently reported [47], however this is unlikely because only virulent strains cause cecum foci, and they yield high levels of cultivable bacteria in feces.

The protective immunity provided by VTnF1 after a six-month period (93% against bubonic plague and 50% against pneumonic plague) is remarkably long-lasting since six months correspond to one third of the mouse life [29], which is comparable to #30 years in human lifespan. Considering that both antibody titers and cellular responsiveness remained high after six months, the almost full protection against bubonic plague may result from either compartment or more likely a synergy of the two [14]. The lower protection against pneumonic plague indicates that lung immunity is the most demanding and requires one or more components of the immune response, that are mandatory for protection but are the first to decline with time. The nature of this component of acquired immunity is yet to determine, but does not seem related to Ig isotype switching or the decline of antibody or IFNγ/IL-17 production. One possible explanation is that aging causes a modification of alveolar macrophages functions, with spontaneous activation and reduced responsiveness to external stimuli, thus contributing to lung susceptibility to infection [48, 49].

Live attenuated *Y*. *pestis* plague vaccines (EV76 and subclones) has been previously used with success in humans, but could generate strong side effects [6, 7], and, as all *Y*. *pestis* strains, are subject to easy genetic rearrangements due to high numbers of insertion sequences in the genome [4, 8]. In addition to genetic stability, VTnF1 combines the known advantages of replicating vaccines: elicitation of humoral and cell-mediated immune responses, robustness against mutant microorganisms, easiness of mass production and use, limited cost, etc., whilst providing guarantees in terms of attenuation, stability, and efficacy against both bubonic and pneumonic plague. Preparedness plans against bioterrorist attacks imply stockpiling millions of vaccine doses. However, stockpiles have a finite lifespan and thus demand regular production of new doses, a rapidly expensive strategy [50]. Live vaccines can be rapidly produced in mass amounts, and are now viewed as a valuable alternative.

In conclusion, we propose here a vaccine providing high-level protection against both bubonic and pneumonic plague after a single-dose immunization. VTnF1 is an easy-to-produce, genetically stable and irreversibly attenuated vaccine, providing a long-lasting and highly efficient protection against both wild type and un-encapsulated (F1-negative) *Y*. *pestis*. To our knowledge, VTnF1 is the only plague vaccine ever reported that could provide high and long lasting protection against both bubonic and pneumonic plague after a single oral administration.

## Supporting Information

S1 Fig Detection of F1 antigen produced by the VTnF1 vaccine strain. To determine the F1 capsule production, a dipstick test devised to detect F1 [21] was used on a cell suspension of the recombinant VTnF1 vaccine strain grown at 37°C in LB broth. The parental V674 strain was used as negative control because it does not possess a *caf* locus, and the *Y*. *pestis* CO92 strain was used as positive control. A positive band at the same position as the *Y*. *pestis* control was observed whereas no signal was detected with the V674 *Y*. *pseudotuberculosis* strain, thus indicating that VTnF1 synthesizes the F1 antigen.
(DOCX)

S2 Fig Follow-up of mice weight after vaccination. Mice were weighed at regular intervals after oral vaccination with VTnF1 (10^8^ CFU) or left unvaccinated (Naïve). Shown are the means of 16 naïve mice and 32 vaccinated mice. Groups were compared at each time point using the unpaired Student’s t test. *: p <0.05; **: p <0.01. ns: not significant.
(DOCX)

S1 Table Bacterial strains and plasmids used in this study. (DOCX)
