# Supplementary material for: Complete Protection against Pneumonic and Bubonic Plague after a Single Oral Vaccination
Source: PLoS Negl Trop Dis. 2015 Oct 16;9(10):e0004162. doi: 10.1371/journal.pntd.0004162 (PMC4608741; doi:10.1371/journal.pntd.0004162)
Supplement: S1 Fig — To determine the F1 capsule production, a dipstick test devised to detect F1 [21] was used on a cell suspension of the recombinant VTnF1 vaccine strain grown at 37°C in LB broth. The parental V674 strain was used as negative control because it does not possess a caf locus, and the Y. pestis CO92 strain was used as positive control. A positive band at the same position as the Y. pestis control was observed whereas no signal was detected with the V674 Y. pseudotuberculosis strain, thus indicating that VTnF1 synthesizes the F1 antigen. (DOCX) [file pntd.0004162.s001.docx]

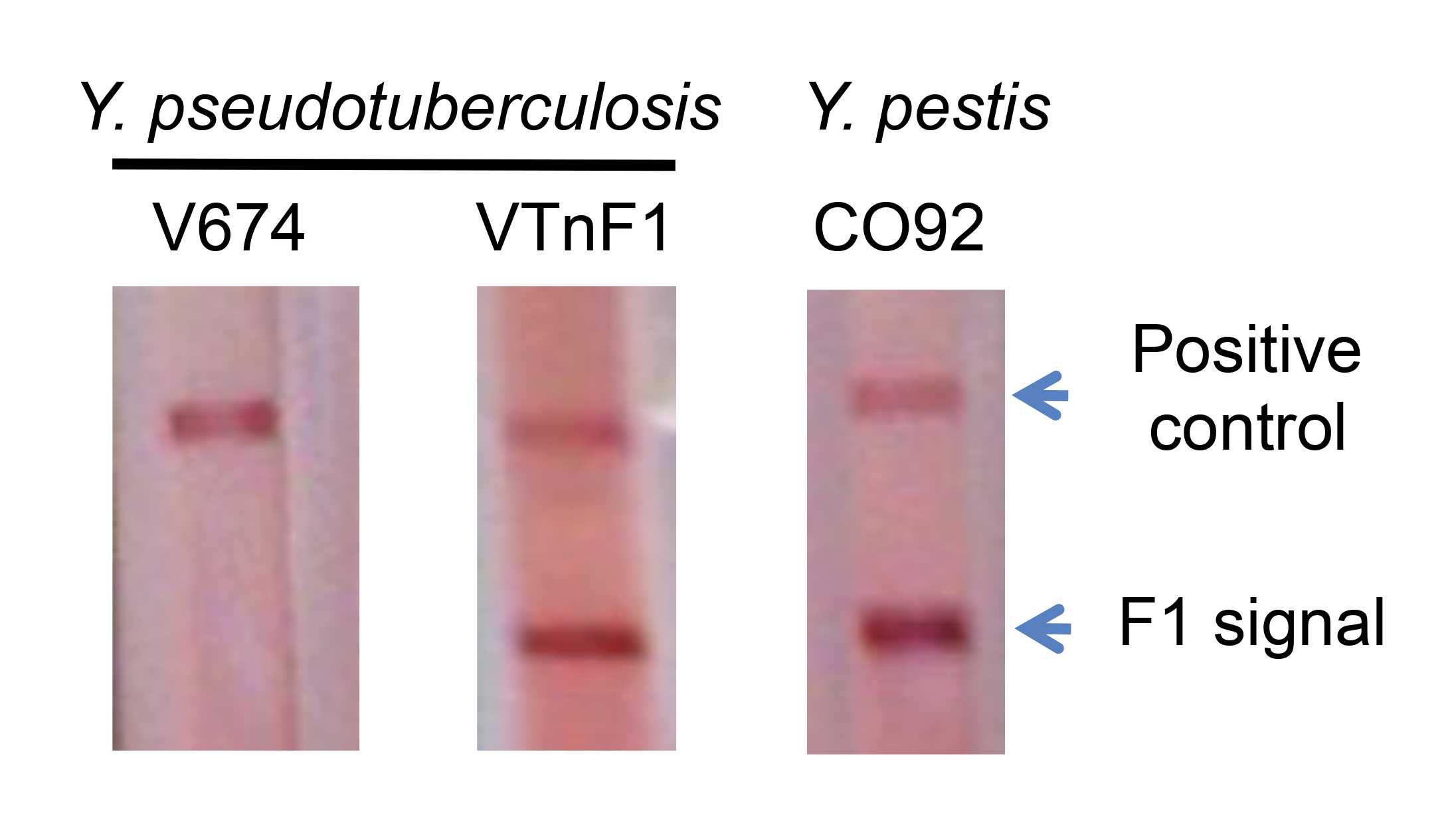


S1 Figure: Detection of F1 antigen produced by the VTnF1 vaccine strain. To determine the F1 capsule production, a dipstick test devised to detect F1 (Chanteau et al. 2003) was used on a cell suspension of the recombinant VTnF1 vaccine strain grown at 37°C in LB broth. The parental V674 strain was used as negative control because it does not possess a *caf* locus, and the *Y. pestis* CO92 strain was used as positive control. A positive band at the same position as the *Y. pestis* control was observed whereas no signal was detected with the V674 *Y. pseudotuberculosis* strain, thus indicating that VTnF1 synthesizes the F1 antigen.

Reference:

Chanteau, S., Rahalison, L., Ralafiarisoa, L., Foulon, J., Ratsitorahina, M., Ratsifasoamanana, L., Carniel, E., and Nato, A. (2003) Development and testing of a rapid diagnostic test for bubonic and pneumonic plague. Lancet 361, 211-216
