# Supplementary material for: Complete Protection against Pneumonic and Bubonic Plague after a Single Oral Vaccination
Source: PLoS Negl Trop Dis. 2015 Oct 16;9(10):e0004162. doi: 10.1371/journal.pntd.0004162 (PMC4608741; doi:10.1371/journal.pntd.0004162)
Supplement: S2 Fig — Mice were weighed at regular intervals after oral vaccination with VTnF1 (108 CFU) or left unvaccinated (Naïve). Shown are the means of 16 naïve mice and 32 vaccinated mice. Groups were compared at each time point using the unpaired Student’s t test. *: p <0.05; **: p <0.01. ns: not significant. (DOCX) [file pntd.0004162.s002.docx]

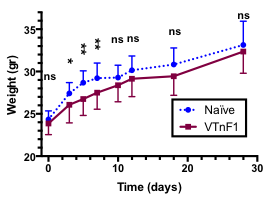


S2 Figure: Follow-up of mice weight after vaccination.

Mice were weighed at regular intervals after oral vaccination with VTnF1 (10^8^ CFU) or left unvaccinated (Naïve). Shown are the means of 16 naïve mice and 32 vaccinated mice. Groups were compared at each time point using the unpaired Student’s t test. *: p <0.05; **: p <0.01. ns: not significant.
