# Supplementary material for: Complete Protection against Pneumonic and Bubonic Plague after a Single Oral Vaccination
Source: PLoS Negl Trop Dis. 2015 Oct 16;9(10):e0004162. doi: 10.1371/journal.pntd.0004162 (PMC4608741; doi:10.1371/journal.pntd.0004162)
Supplement: S1 Table — (DOCX) [file pntd.0004162.s003.docx]

**S1 Table :** Bacterial strains and plasmids used in this study

|  |  | **Characteristics** | **Origin** |
| --- | --- | --- | --- |
| ***Y. pestis*** |  |  |  |
| CO92 |  | Wild type, biotype Orientalis | ([30](#_ENREF_30)) |
| CO92∆*caf* |  | CO92 deleted of the *caf* operon | ([15](#_ENREF_15)) |
| CO92::Tn7-P*ail*-*lux* |  | Tn7-P*ail-lux* integrated into the chromosomal att-  Tn*7* site of CO92 | This study |
| ***Y. pseudotuberculosis*** |  |  |  |
| V674 |  | IP32953p strain deleted of the HPI, *yopK* and *psaA*  genes | ([15](#_ENREF_15)) |
| V674pF1 |  | pGEN-*caf* plasmid introduced into V674 by  electroporation | ([15](#_ENREF_15)) |
| VTnF1 (V674::Tn7*caf*-CmR-FRT) |  | Mini-Tn7*caf* integrated into the chromosomal att-Tn*7* site of V674 | This study |
| **Plasmids** |  |  |  |
| pFCM1 |  | CmR-FRT cassette vector | ([16](#_ENREF_16)) |
| pFKM1 |  | KmR-FRT cassette vector | ([16](#_ENREF_16)) |
| pGEN-*lux* |  | *luxCDABE* operon from *Photorabdus luminescens* | ([17](#_ENREF_17)) |
| pUC18R6KTn*7*-Cm^R^ |  | Suicide mini-Tn*7*-Cm^R^ delivery vector | This study |
| pUC18R6KTn*7*-Cm^R^-*caf* |  | Suicide mini-Tn*7*-Cm^R^-*caf* delivery vector  *(caf* operon cloned into the *Apa*I/*EcoR*I sites of  pUC18R6KTn*7*-Cm^R^) | This study |
| pUC18R6KTn*7*-Km^R^ |  | Suicide mini-Tn*7*-Km^R^ delivery vector | This study |
| pUC18R6KTn*7*-Km^R^-*lux* |  | Suicide mini-Tn*7*-Km^R^-P*ail*-*lux* delivery vector | This study |
| pTSN2 |  | Tn*7* transposase expression vector, not replicative in *Yersinia* | ([16](#_ENREF_16)) |
